# Leigh-like syndrome with mild mtDNA depletion due to the *SUCLG1* variant c.626C > T

**DOI:** 10.1016/j.ymgmr.2018.12.001

**Published:** 2018-12-06

**Authors:** Josef Finsterer

**Affiliations:** Krankenanstalt Rudolfstiftung, Messerli Institute, Veterinary University of Vienna, Postfach 20, 1180 Vienna, Austria

**Keywords:** Mtdna, Depletion, Metabolic myopathy, Brain, Leigh syndrome, suclg1, Respiratory chain

Letter to the Editor

With interest we read the case report by Chinopoulos et al. about a 3.5 years old female with fatal encephalopathy cataract, hypoacusis, and myopathy due to the heterozygous mutation c.626C > A in the *SUCLG1* gene with mtDNA depletion to 65% in muscle [1]. There are a number of shortcomings.

It is contradictory that the index patient initially presented with general hypotonia but exaggerated deep tendon reflexes. Simultaneous spasticity and hypotonia requires explanation.

The twin sister of the index case died from late intrauterine death 10 days prior to birth [1]. We should know if the twin sister presented with any phenotypic or genotypic features of the index case and if autopsy indicated mitochondrial depletion syndrome.

The authors state that respiratory chain complex functions in skin fibroblasts were normal but table 3 indicates that activity of complex-I was not measured and that combined complex-II/III activity was reduced [1]. This discrepancy requires an explanation.

The patient was described as functionally homozygous [1] but was not investigated for intronic mutations in the non-mutated allele [2].

The discrepancy between mild mtDNA depletion in muscle and the severe phenotype remains unexplained. Possibly, mtDNA depletion was more pronounced in tissues clinically more severely affected than muscle, like the brain [3]. The amount of mtDNA depletion in the brain at autopsy would be interesting.

The index patient's father also carries the c.626C > A variant [1]. Surprisingly, he was phenotypically unaffected. The mismatch between the same genotype of father and daughter but dissimilar phenotype requires explanation.

Overall, this interesting case report could be more meaningful if respiratory chain complex functions and the degree of mtDNA depletion would have been determined in the brain, if the *SUCLG1* gene would have been examined also for intronic variants, and if reduction and mislocalisation of SUCLG2 would have been more conclusively explained.
